# LeafSightX: an explainable attention-enhanced CNN fusion model for apple leaf disease identification

**DOI:** 10.3389/frai.2025.1689865

**Published:** 2026-01-30

**Authors:** Md. Ehsanul Haque, Fahmid Al Farid, Md. Kamrul Siam, Md. Nurul Absur, Jia Uddin, Hezerul Abdul Karim

**Affiliations:** 1 Department of Computer Science and Engineering, East West University, Dhaka, Bangladesh; 2 Centre for Image and Vision Computing (CIVC), Faculty of Artificial Intelligence and Engineering, Multimedia University, Cyberjaya, Malaysia; 3 Department of Computer Science, New York Institute of Technology, New York, NY, United States; 4 Department of Computer Science, City University of New York, New York, NY, United States; 5 AI and Big Data Department, Woosong University, Daejeon, Republic of Korea

**Keywords:** apple leaf disease, convolutional neural networks (CNNs), DenseNet201, explainable artificial intelligence (XAI), InceptionV3, multi-head self-attention (MHSA), plant disease detection, precision agriculture

## Abstract

The rapid and precise identification of apple leaf diseases is crucial for minimizing yield loss in precision agriculture. However, many existing deep learning methods struggle to be applicable in real-world settings, are not easily interpretable, and often lack sufficient statistical validation. To address these difficulties, we propose our solution approach *LeafSightX*. This dual-backbone architecture combines features from DenseNet201 and InceptionV3 using Multi-Head Self-Attention (MHSA) techniques, enhancing representational capability and spatial context reasoning. Our extensive procedure includes specialized preprocessing and limited data augmentation, improving model resilience in many scenarios. Furthermore, *LeafSightX* integrates explainable AI techniques with Grad-CAM visualizations to improve transparency. In assessments of a five-class apple leaf disease dataset featuring field and laboratory images, LeafSightX demonstrates exceptional performance, attaining a test accuracy of 99.64%, an F1-score of 0.9962, and AUC and PR-AUC scores of 1.000, far surpassing all baseline CNNs. Cross-validated Cohen's Kappa (mean = 0.9917, σ = 0.0020) and AUC (mean = 0.9998) indicate a significant level of predictive consistency. Despite its architectural complexity, the model offers real-time inference capabilities, ensuring per-sample latency suitable for edge device deployment. Additionally, the proposed LeafSightX framework was trained and evaluated on an additional independent apple leaf disease dataset, achieving a test accuracy of 99.69%, demonstrating its robustness and generalization. Our approach is a rigorously evaluated, clear, and highly accurate system for identifying plant diseases, providing a reproducible foundation for the actual application of AI in agriculture.

## 1 Introduction

Agriculture plays a vital role in ensuring global food security and maintaining economic stability (Aboelenin et al., 2025). Apple *(Malus domestica)* is of significant commercial importance among fruit crops due to its nutritional value and market demand. Nonetheless, the production and quality of apples are severely threatened by several foliar diseases, including Alternaria leaf spot, rust, gray spot, and brown spot (Cabrefiga et al., 2023). These diseases result in significant yield losses and directly influence fruit appearance and quality, causing severe economic harm (Hussain, 2024; Vurro et al., 2010; Bonkra et al., 2024). Early and precise diagnosis of apple leaf diseases is thus critical for sustainable production and disease management (Fadia et al., 2019).

**Figure 1:**
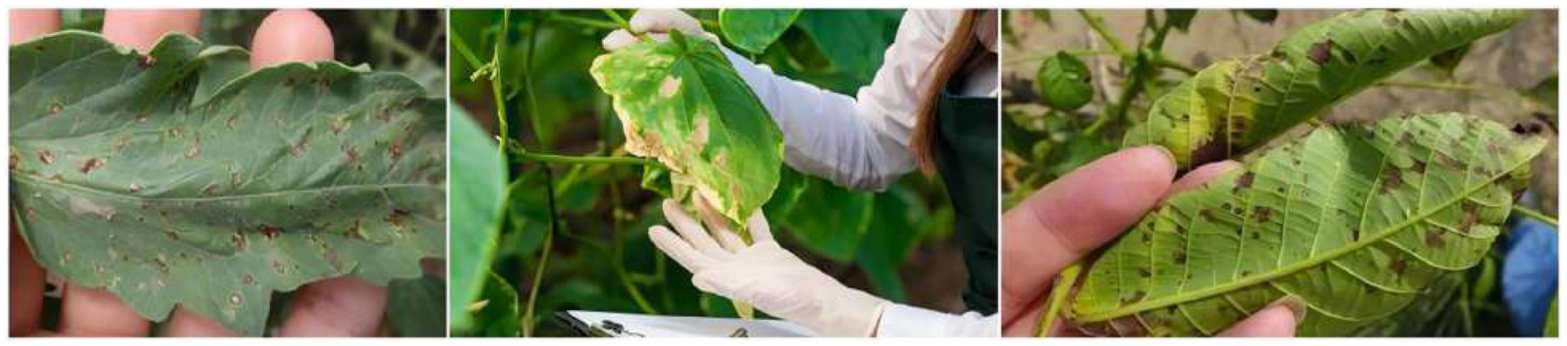
Manual inspection of leaves by an agricultural expert in an orchard setting. The process is time-consuming and subjective, motivating the need for automated computer vision systems (Fu et al., 2025).

Historically, farmers and agricultural specialists have used manual inspection to detect the disease on apple leaves (Khan et al., 2022). This technique is based on optical observation of leaf surfaces, during which any visible lesions, discoloration, fungal plaques, or necrotic tissue are assessed. Despite its simplicity and widespread use, manual diagnosis is a slow, labor-intensive, and highly expert, experience-based method. In addition, accuracy is often diminished by subjective judgment, fatigue, and environmental factors such as changes in lighting. Manual inspection is inefficient in large-scale orchards or rural settings, where qualified experts are scarce and making timely decisions is difficult. Figure 1 illustrates the manual examination process performed by an agricultural expert in an orchard environment.

Due to the rapid advancement of computer vision and deep learning, automated plant disease diagnosis has become a compulsory thing (Shafay et al., 2025; Upadhyay et al., 2025). VGG19, InceptionV3, DenseNet201, and Xception are convolutional neural network architectures that have demonstrated impressive results in image-based classification. Nevertheless, even with these developments, the current practices continue to encounter significant challenges. It is common in many models to have limited generalization to actual field images that do not match laboratory conditions. Interpretability also tends to be lacking in most systems, and it is hard to explain why they make the predictions they do (Doutoum and Tugrul, 2023). Moreover, the inability to use reliable probability calibration can lead to overconfident or underconfident decisions, thereby adversely affecting the practice of precision agriculture. Also, Computational cost analysis is rarely discussed in existing studies on plant disease detection. Yet, it plays a critical role in determining the real-time applicability and practicality of deep learning models in agricultural environments.

To address these drawbacks, a robust, explainable deep learning model that provides reliable diagnoses across different cases is an immediate necessity. To meet this requirement, we present LeafSightX, a more sophisticated system for automatically tracing and identifying apple leaf diseases. LeafSightX combines the backbones of DenseNet201 and InceptionV3 via a Multi-Head Self-Attention mechanism, enabling the model to remember both local texture features and wide contextual features. A full preprocessing pipeline of Gaussian blur filtering, contrast-limited adaptive histogram equalization, and intensity normalization is used to maximize image quality and minimize environmental variation. Also, Grad CAM visualization is added to highlight the disease area, making models more transparent and interpretable.

The contribution of this study is summarized as:

- LeafSightX is a new deep learning framework that combines DenseNet201 and InceptionV3 with Multi-Head Self-Attention to capture both small details and overall patterns for accurate apple leaf disease detection.
- A robust preprocessing pipeline using Gaussian blur, contrast adjustment, and normalization improves image quality and handles changes in light and environment.
- Explainable artificial intelligence using Grad-CAM highlights the leaf regions that influence predictions, making the results easier for experts to understand.
- Reliability and computational cost are evaluated using Brier Score, permutation testing, bootstrap confidence intervals, Cohen Kappa, and inference time, addressing the rare focus on efficiency for real-time deployment.
- LeafSightX outperforms existing methods in accuracy, reliability, and efficiency, providing more trustworthy results.
- The framework is validated on an additional dataset to demonstrate robustness and generalization to new images and conditions.

The recognized constraints would be mitigated by the proposed conceptual design, which aims to provide an automated leaf disease detection system that serves as a replicable, interpretable, and efficient tool for precision agriculture and sustainable apple growing worldwide.

The subsequent sections of the paper are organized as follows: Section 2 presents a literature review on the identification of apple leaf diseases using deep learning techniques. Section 3 describes the proposed LeafSightX, the dataset pre-processing, and the training environment. The experimental results and model interpretability are explored in Section 4 with the aid of Grad-CAM visuals. Finally, Section 5 summarizes the article and provides guidance for further research.

## 2 Literature review

The precise, effective, and resilient identification of apple leaf diseases has been a fundamental challenge in precision agriculture, and recent advancements in deep learning have been a significant catalyst in addressing it. A-Net, proposed by Liu and Li (2024), was a YOLOv5-based framework that incorporated an additional Wise-IoU loss function and RepVGG modules. This model exhibited an exceptional detection rate, with a mean average precision (mAP@0.5) of 92.7%. However, its adaptability to diverse environmental conditions had not been examined. Wang et al. (2025) presented ELM-YOLOv8n, which integrated Efficient Multi-Scale Attention (EMA) and DESCS-DH blocks to balance speed and accuracy while maintaining a lightweight design. It achieved a mAP@0.5 of 96.7% and an F1-score of 94.0%, but still faced challenges in real-time applicability and robustness across heterogeneous datasets. Similarly, Gong and Zhang (2023) employed GhostConv and GAM modules in a compact YOLOv8 variant, attaining a mAP@0.5 of 86.9% with very few parameters; however, concerns remained regarding the model's generalizability and explainability.

Similarly, Lv and Su (2024) applied transformer encoders and CBAM in YOLOv5-CBAM-C3TR to improve feature description. Although it achieved 92.4% accuracy, it was limited by its inability to incorporate newer YOLO variants (YOLOv7/YOLOv8). Scientific (2025) proposed a VGG-DAGSVM model that employed bilateral filtering and SegNet-based segmentation. While it achieved 96.5% classification accuracy, the robustness of its preprocessing and segmentation methods was unsatisfactory. Moreover, Luo (2025) introduced AppleLite-YOLOv8, which integrated EdgeNeXt and C2f-SC modules. This system achieved 97.56% accuracy and 94.38% recall, yet struggled in complex backgrounds and uneven lighting.

In a recent work, Rajput et al. (2024) implemented Neutrosophic Logic with EfficientNetB0 to address uncertainty, reporting an accuracy of 99.51%. Nevertheless, the lack of cross-validation and interpretability reduced its reliability. Segmentation-integrated approaches also gained traction. Parashar and Johri (2024) achieved an accuracy of 94.76% by incorporating Canny edge detection and watershed transformation into a CNN framework. However, the segmentation methods were conventional, offered no explainability, and were not validated. Rohith et al. (2025) compared CNNs including ResNet, VGG19, and InceptionV3. When combined with ResNet, VGG19, and InceptionV3, the models achieved maximal validation accuracies of 98.9%, 97.1%, and 97.4%, respectively. However, these studies lacked robustness testing and interpretability.

In contrast, Liu et al. (2024) proposed MCDCNet, which combined multi-scale fusion with constrained deformable convolution to enhance geometric adaptability. It demonstrated a detection accuracy of 66.8% and performed well at capturing spatial deformations, but failed to stand out in identifying overlapping lesions. Furthermore, Zhang et al. (2023) introduced BCTNet, which incorporated a Bole Convolution Module and bidirectional feature fusion. The model achieved 85.23% accuracy and a real-time inference rate of 33 FPS, but its effectiveness was limited by dataset diversity. Khan et al. (2022) proposed a two-step lightweight classification and symptom localization network, trained on 9,000 RGB images, achieving 88% classification accuracy and 42% mAP. Despite its ability to operate in real time, the dataset was limited in diversity, scalability was questionable, and the network misclassified subtle symptoms. It achieved a recall of 49.0% and a mean Average Precision of 34.0%. Although it performed reasonably well in complex environments, it remained prone to misclassifying small lesions and did not generalize across datasets.

Despite the increasing number of studies on apple leaf disease detection, several critical gaps persisted. First, the generalizability of the findings was limited by the lack of replication and validation methods, particularly cross-validation, which involves dividing the data into training and test sets to train and test models multiple times. Second, most studies employed segmentation and preprocessing algorithms (steps to highlight affected leaf areas and prepare data before analysis) that were either inadequately optimized or simplistic, rendering them ineffective in isolating complex or overlapping lesions (Sharma et al., 2020; Dayang and Meli, 2021). Third, explainability, meaning the ability to interpret how and why a model makes decisions, was often absent, which undermined trust in model predictions. Fourth, although models demonstrated high accuracy under controlled conditions, few were rigorously tested across varying environmental conditions, such as lighting, background clutter, or leaf orientation, thereby limiting their field applicability. Moreover, computational expense, referring to the amount of computing resources required, remained high due to complex architectures with numerous parameters. Yet, this issue was seldom quantified or analyzed, raising concerns about deployment on resource-constrained devices. Finally, recent research shows that insufficient application of statistical validation methods, such as analysis of variance (ANOVA) and t-tests, compromised the empirical rigor and robustness of the reported findings (Saho, 2025).

These shortcomings underscore the need for future research to prioritize computational efficiency, statistically rigorous validation, advanced preprocessing, and explainability. Addressing these gaps would improve the practical applicability, reliability, and portability of automated apple leaf disease detection systems in precision agriculture. This paper addressed these gaps by introducing a comprehensive preprocessing strategy and extensive data augmentation to improve model resilience. A novel dual-backbone transfer learning framework, *LeafSightX*, was developed, integrating MHSA operations for enhanced feature representation and classification accuracy. The model was validated through cross-validation and statistical testing to confirm the significance of improvements. Furthermore, XAI methods were incorporated to ensure interpretability and transparency. Finally, the practicality and generalizability of the system were enhanced by training on both laboratory-controlled and field-collected images under varied environmental conditions, while substantially reducing inference time per sample for real-time agricultural deployment.

## 3 Methodology

**Figure 2:**
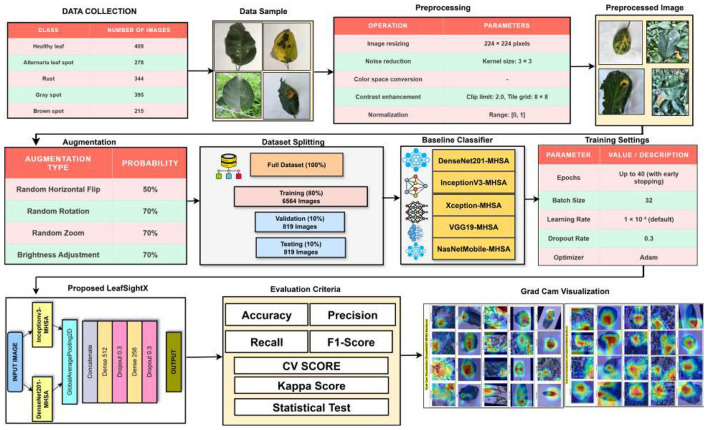
Workflow diagram of proposed Apple leaf disease detection framework.

The proposed methodology for detecting apple leaf disease employs a systematic pipeline aimed at effectively identifying and classifying diverse apple leaf states. The framework's workflow is illustrated in Figure 2. The primary phases of this methodology encompass data gathering, preprocessing and augmentation, disease diagnosis, baseline and suggested model building, training, and performance evaluation.

### 3.1 Data acquisition

This research utilizes the Apple Tree Leaf Disease dataset, collected from Kaggle and made available by Nirmal (Kaggle, 2025). The dataset consists of images of diseased apple leaves gathered from four locations at China's Northwest University of Agriculture and Forestry Science and Technology. Images were taken with a Glory V10 mobile phone in diverse environmental settings, with approximately 52% captured in a controlled laboratory and 48% in natural growing fields. To evaluate the effectiveness of our approach, we also use an additional dataset (Dhar, 2023).

**Figure 3:**
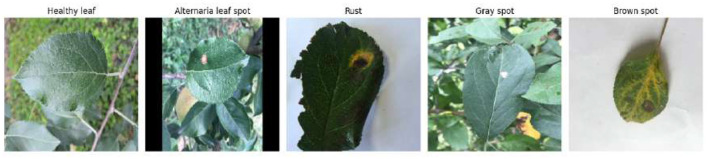
Sample images from the apple leaf disease dataset.

The dataset is divided into five categories: healthy leaf, alternaria leaf spot, rust, gray spot, and brown spot. The class distribution is presented in Table 1, while Figure 3 shows typical sample images from each category.

**Table 1:** Class distribution of the apple leaf disease dataset.

**Class**	**Number of images**
Healthy leaf	409
Alternaria leaf spot	278
Rust	344
Gray spot	395
Brown spot	215
Total	1,641

### 3.2 Image processing and feature enhancement

The image quality was enhanced, and regions of the image associated with disease were highlighted by applying a standard preprocessing pipeline before training the model (Gudge et al., 2025). To ensure the model input was consistent, the first step was to scale the pictures to a specific resolution. Subsequently, a Gaussian blur filter was applied to mitigate high-frequency noise and sensor artifacts, thereby facilitating computation and enabling the model to focus on relevant patterns rather than noise (Gedraite and Hadad, 2011). Also, CLAHE (Contrast Limited Adaptive Histogram Equalization) was applied to the V channel of the HSV color space (Pizer et al., 1990). The step will help with changes in lighting and improve local contrast, making it easier to find illness spots and lesions in images shot under diverse lighting and weather conditions. Finally, the pixel intensities were normalized to a comparable range. Collectively, these preprocessing steps stabilized and optimized the training process, allowing the neural network to learn more effectively and generalize better to heterogeneous data. Overall, such comprehensive preprocessing enhances the reliability and accuracy of the disease classification model. Table 2 summarizes the key preprocessing steps along with their corresponding parameters.

**Table 2:** Preprocessing operations and parameters applied to the apple leaf images.

**Operation**	**Description**	**Parameters**
Image resizing	Uniform scaling to fixed size	224 × 224 pixels
Noise reduction	Gaussian blur smoothing	Kernel size: 3 × 3
Color space conversion	BGR to HSV and back	-
Contrast enhancement	CLAHE applied on Value channel	Clip limit: 2.0, Tile grid: 8 × 8
Normalization	Scaling pixel intensities	Range: [0, 1]

**Figure 4:**
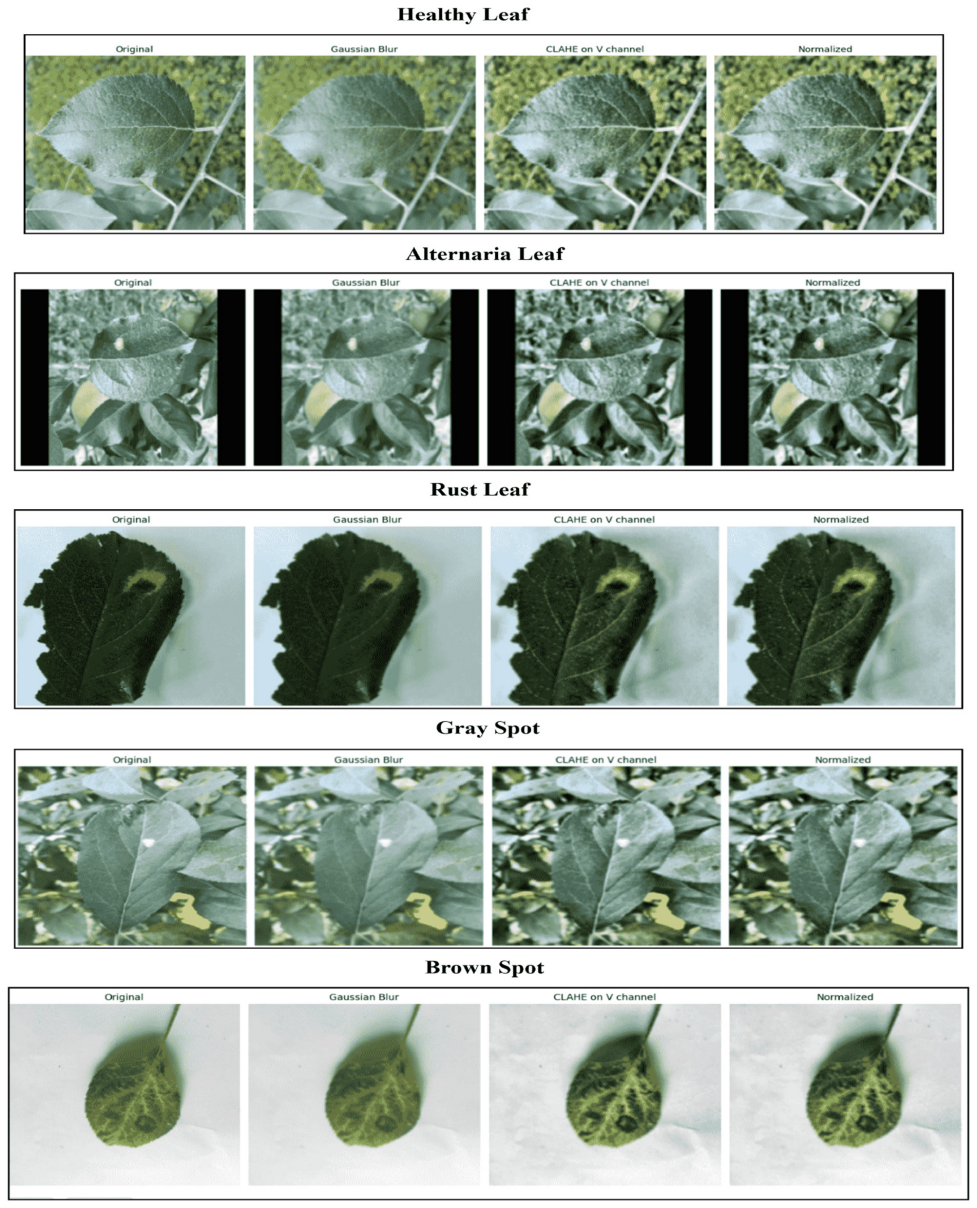
Representative images from the preprocessing pipeline: original, Gaussian blurred, CLAHE-enhanced, and normalized images from **left to right**.

While, Figure 4 shows example images at various stages of the preprocessing pipeline, illustrating the effects of each operation.

### 3.3 Data augmentation

To enhance the dataset's diversity and improve the model's generalization, data augmentation was implemented during preprocessing of Such augmentations reproduce various real-life variations, including orientational, scale, and light variations (Mikolajczyk and Grochowski, 2018; Kumar et al., 2024). Random horizontal flipping helps the model learn to be invariant to leaf orientation. Random rotation enables the capture of leaves at different angles in an image. Random zoom changes the camera's distance, and brightness adjustment adjusts the illumination. Collectively, such transformations make the data sets more variable, thereby contributing to a model that is more robust to varying input states. The augmentation techniques and the parameters are summarized in Table 3.

**Table 3:** Data augmentation techniques and parameters.

**Augmentation type**	**Description**	**Probability**
Random horizontal flip	Flips the image horizontally	50%
Random rotation	Rotates image randomly (–30° to +30°)	70%
Random zoom	Zooms image randomly (80%–120% of original size)	70%
Brightness adjustment	Scales brightness (60%–140% of original)	70%

This augmentation multiplied the size of the dataset five times the initial reading of five samples, each containing one original image and four variants of augmentation. As a result, the augmented image counts per class are shown in Table 4.

**Table 4:** Number of images per class after augmentation.

**Class**	**Number of images**
Healthy leaf	2,045
Alternaria leaf spot	1,390
Rust	1,720
Gray spot	1,975
Brown spot	1,075
**Total**	**8,205**

### 3.4 Dataset splitting

The augmented data set was divided into training, validation, and testing sets with ratios of 80%, 10%, and 10%, respectively. This division is typically applied to ensure that the model used has sufficient data to learn from during training, and that there is enough data to verify model execution and ultimately test it (Mamun et al., 2025). The division was conducted on a class basis to ensure proportional representation in all subsets. Table 5 contains the summary of the split number of images per class.

**Table 5:** Dataset split summary (number of images per class).

**Class**	**Train**	**Validation**	**Test**
Healthy leaf	1,636	204	205
Alternaria leaf spot	1,112	139	139
Rust	1,376	172	172
Gray spot	1,580	197	198
Brown spot	860	107	108
**Total**	6,564	819	822

### 3.5 Baseline models

This study utilizes five state-of-the-art convolutional neural networks, namely DenseNet201, InceptionV3, VGG19, NASNetMobile, and Xception, as the baseline to investigate the classification of diseases in apple leaves. All the models used were optimized to include an MHSA mechanism with four attention heads, which better represent their features and capture long-range dependencies.

#### 3.5.1 Densenet201-MHSA

The DenseNet201 architecture comprises 201 layers of batch normalization and 201 layers of activation, inserted between 200 convolutional (Conv2D) layers, enabling the extraction and reuse of features through dense block-wise connectivity. They are then sequentially merged by 98 concatenation layers that enable the network to retain information across layers and integrate it. The spatial dimensions are progressively suppressed through three average pooling layers and one single max pooling layer. At the same time, the feature maps are zero-padded at two pooling levels to preserve their size. A flat layer follows the feature extraction, and then the three dense layers perform the final classification. There are two dropout layers to minimize overfitting, and two lambda layers have custom tensor operations. This architecture consists of a single input embedding layer, a mid-layer MHSA, and four-head self-attention after the convolutional blocks to learn long-range spatial correlations. Such an attention level is accompanied by a one-layer normalization and an additional layer, thereby establishing residual connections that improve learning stability and representation. Combined, these elements allow DenseNet201-MHSA to capture intricate patterns in the images of stable apple leaves with diseases. To describe the structural composition of the DenseNet201-MHSA architecture on a quantitative level, the frequency and structure of its primary type of layers are outlined in Table 6.

**Table 6:** Layer-wise summary of the DenseNet201-MHSA architecture.

**Layer type**	**Output shape**	**Description**
Input	224 × 224 × 3	RGB image input
Conv2D (3 × 3, s=2)	112 × 112 × 64	Initial feature extractor
BatchNorm + ReLU	112 × 112 × 64	Normalization and activation
Conv2D × 200	Varies	Dense convolutional units
BatchNorm × 201	Varies	Applied post-conv
ReLU × 201	Varies	Non-linearity
Concatenate × 98	Varies	Dense feature reuse
MaxPool2D × 1	56 × 56 × *	Downsampling
AvgPool2D × 3	Varies	Spatial reduction
ZeroPad2D × 2	Varies	Size preservation
GlobalAvgPool2D	1 × 1 × *C*	Vector output
Lambda × 2	(*B, S, E*)	Tensor reshape
MHSA	(*B, S, E*)	Self-attention
LayerNorm	(*B, S, E*)	Normalize features
Add	(*B, S, E*)	Residual link
Dense (512)	(*B*, 512)	Feature projection
Dropout × 2	(*B*, ·)	Regularization
Dense (256)	(*B*, 256)	Abstraction
Dense (Classifier)	(*B*, 1285)	Class logits
Softmax	(*B*, 1285)	Probability output

#### 3.5.2 InceptionV3-MHSA

InceptionV3-MHSA is an architecture that begins with a single input layer, followed by 94 convolutional layers, coupled with batch normalization and activation layers, to facilitate balance and speed during training. It features four max pooling layers and nine average pooling layers, which can progressively downsample the spatial dimension while preserving essential features. The 15 concatenated layers interconnect the inception modules, enabling multi-scale feature learning across various convolutional branches. The spatial dimensions are flattened into a compact feature vector, which is then fed into three dense layers, followed by a final classification layer with average pooling (also known as global average pooling). To avoid overfitting, dropout is applied twice per layer, whereas two lambda layers can be used to apply customized tensor operations. A layer normalization layer has been added to improve stability during training and to provide consistent feature scaling across four attention heads. An MHSA layer has been added before the final dense layers to improve spatial feature representation. Such an arrangement enables InceptionV3-MHSA to successfully learn local and global patterns in the apple leaf dataset. Table 7 presents the key layer summary of the InceptionV3-MHSA architecture.

**Table 7:** Layer-wise summary of the InceptionV3-MHSA architecture.

**Layer type**	**Output shape**	**Description**
Input	299 × 299 × 3	RGB image input
Conv2D (3 × 3)	149 × 149 × 32	Edge extraction
Conv2D (3 × 3)	147 × 147 × 32	Spatial refinement
Conv2D (3 × 3)	147 × 147 × 64	Feature expansion
MaxPooling2D	73 × 73 × 64	Downsampling
Conv2D (1 × 1)	73 × 73 × 80	Channel projection
Conv2D (3 × 3)	71 × 71 × 192	Depth increase
MaxPooling2D	35 × 35 × 192	Further reduction
Mixed0 (Incep-A)	35 × 35 × 256	Multi-branch features
Mixed1 (Incep-A)	35 × 35 × 288	Expanded features
Mixed2 (Incep-A)	35 × 35 × 288	Repeat extraction
Mixed3 (Incep-B)	17 × 17 × 768	Deeper abstraction
Mixed4–7	17 × 17 × 768	Inception-B stack
Mixed8 (Incep-C)	8 × 8 × 1280	Wider features
Mixed9–10	8 × 8 × 2048	Final feature blocks
GlobalAvgPool2D	1 × 1 × 2048	Vector output
Dense	(*B*, 1, 285)	Class logits
Dropout	(*B*, 1, 285)	Regularization
Lambda	(*B, S, E*)	Reshape for attention
MHSA	(*B, S, E*)	Self-attention
LayerNorm	(*B, S, E*)	Feature normalization
Add	(*B, S, E*)	Residual link
Dense (FFN)	(*B*, 512)	Intermediate projection
Dropout	(*B*, 512)	Regularization
Dense (classifier)	(*B*, 1, 285)	Final output

#### 3.5.3 VGG19-MHSA

The VGG19-MHSA model architecture begins with a single input layer, followed by 16 successive convolutional layers that generate hierarchical features from the input image. These are interspersed with five max pooling layers to compact the spatial dimensions while preserving essential structures. There is a global average pooling layer in between, which reduces the spatial features to a small representation after feature extraction. It goes through a triplet-dense layer, with two injection dropout layers to help prevent overfitting and improve generalization. A MHSA layer, consisting of four attention heads, is added to enable the model to focus on various regions of space and perform global contextual reasoning. A layer normalization layer will be used to stabilize the attention output, and two lambda layers will be employed to inject custom operations, enabling greater architectural flexibility. The features with attention are brought to an earlier representation via an additional layer, enriching the final classification pathway. Table 8 presents the key layer summary of the VGG19-MHSA architecture.

**Table 8:** Layer-wise summary of the VGG19-MHSA architecture.

**Layer type**	**Output shape**	**Description**
Input	224 × 224 × 3	RGB image input
Block1_Conv1	224 × 224 × 64	3 × 3 conv layer
Block1_Conv2	224 × 224 × 64	3 × 3 conv layer
Block1_Pool	112 × 112 × 64	2 × 2 max pool
Block2_Conv1	112 × 112 × 128	3 × 3 conv
Block2_Conv2	112 × 112 × 128	3 × 3 conv
Block2_Pool	56 × 56 × 128	2 × 2 max pool
Block3_Conv1–4	56 × 56 × 256	4 conv layers
Block3_Pool	28 × 28 × 256	2 × 2 max pool
Block4_Conv1–4	28 × 28 × 512	4 conv layers
Block4_Pool	14 × 14 × 512	2 × 2 max pool
Block5_Conv1–4	14 × 14 × 512	4 conv layers
Block5_Pool	7 × 7 × 512	2 × 2 max pool
GlobalAvgPool2D	1 × 1 × 512	Vector flattening
Dense	(*B*, 1, 285)	Fully connected layer
Dropout	(*B*, 1, 285)	Regularization
Lambda	(*B, S, E*)	Reshape for attention
MHSA	(*B, S, E*)	Self-attention
LayerNorm	(*B, S, E*)	Normalize output
Lambda	(*B, S, E*)	Tensor reshape
Add	(*B, S, E*)	Residual connection
Dense (FFN)	(*B*, 512)	Intermediate projection
Dropout	(*B*, 512)	Regularization
Dense (Output)	(*B*, 1285)	Final logits

#### 3.5.4 Xception-MHSA

The Xception-MHSA has a single input layer, and the remaining layers comprise 116 convolutional layers that use depthwise separable convolutions to learn fine spatial features at low resolution automatically. This has been accompanied by 126 batch normalization layers and 126 activation functions, which enhance both nonlinearity and learning stability. It is done through max pooling, and the feature maps are down-sampled three times. It also has nine average pooling layers, and the final layer is a global average pooling layer that reduces the spatial dimensions to a feature vector. The network has three dense layers for final prediction, with two dropout layers in between for regularization. A Multi-Head Self-Attention Mechanism is added to increase feature interaction across the globe, utilizing four heads. Layer normalization is then applied to stabilize the attention output process. Custom computation is implemented across two lambda layers, and 24 add layers are strategically distributed to combine intermediate representations, thereby maintaining information flow through residual connections. The last representation is detailed not only in the local context but also in the international context. Table 9 presents the key layer summary of the Xception-MHSA architecture.

**Table 9:** Layer-wise summary of the Xception-MHSA architecture.

**Layer type**	**Output shape**	**Description**
Input	224 × 224 × 3	RGB image input
Block1_Conv1	112 × 112 × 32	Entry conv
+ BN + ReLU	112 × 112 × 32	Normalize + activate
Block1_Conv2	112 × 112 × 64	Entry conv
+ BN + ReLU	112 × 112 × 64	Normalize + activate
Block2_SepConv1–2	56 × 56 × 128	Entry separable convs
+ Pool + Add	56 × 56 × 128	Downsample + residual
Block3 ( × 2)	28 × 28 × 256	Middle separable + residual
Block4 ( × 2)	14 × 14 × 728	Deep separable convs
Block5–12 ( × 8)	14 × 14 × 728	Repeated convs + residuals
Block13	14 × 14 × 1024	Final middle conv
+ Pool + Add	7 × 7 × 1024	Downsample + residual
Block14	7 × 7 × 2048	Exit flow conv
GlobalAvgPool2D	1 × 1 × 2048	Feature vector
Dense	(*B*, 1285)	Class projection
Dropout	(*B*, 1285)	Regularization
Lambda	(*B, S, E*)	Reshape for attention
MHSA	(*B, S, E*)	Self-attention layer
LayerNorm	(*B, S, E*)	Normalize features
Lambda	(*B, S, E*)	Reshape for FFN
Add	(*B, S, E*)	Residual skip connection
Dense (FFN)	(*B*, 512)	Intermediate dense
Dropout	(*B*, 512)	Regularization
Dense (Output)	(*B*, 1285)	Final logits

#### 3.5.5 NasNetMobile-MHSA

The NASNetMobile-MHSA model comprises a model framework that begins with an input layer, followed by a composition of 36 conventional convolutional layers and a massive 160 separable convolutional layers, all parametrized to be space-aware. All convolutions are followed by the same number of batch normalization layers and activation layers, with 192 per instance, to train the networks to greater depth and learn non-linear representations. All eight max pooling and 52 average pooling layers are used to perform the spatial reduction and refinement. It features a zero-padding and cropping architecture, utilizing 24 zero-padding and 4 cropping layers, respectively, to adjust the parameter input dimensions to fit the desired dimensions while preserving edge information. The network extensively utilizes 20 concatenation layers and 81 addition layers to reduce the number of features across branches for combination. After the feature maps, they undergo global average pooling and are fed into three dense layers for classification, with two dropout layers introduced to curb overfitting. There is an MHSA layer with four heads, which enables modeling of global context, and a layer normalization layer that stabilizes the training process. Two lambda layers also enable custom tensor operations, making the architecture even more flexible. Table 10 shows the key layer summary of the NasNetMobile-MHSA architecture.

**Table 10:** Layer-wise summary of the NasNetMobile-MHSA architecture.

**Layer type**	**Output shape**	**Description**
Input	224 × 224 × 3	RGB image input
Stem_Conv1	112 × 112 × 32	Initial conv layer
BN + ReLU	112 × 112 × 32	Normalization + activation
SepConv1	56 × 56 × 64	Depthwise separable conv
SepConv2	56 × 56 × 128	Feature expansion
MaxPool1	28 × 28 × 128	Downsampling
SepConv3—4	28 × 28 × 256, 512	Deeper conv layers
SepConv5	14 × 14 × 512	Further depth
MaxPool2	14 × 14 × 512	Downsampling
SepConv6	7 × 7 × 1, 024	Final conv output
GlobalAvgPool	1 × 1 × 1024	Feature flattening
Dense1	(*B*, 1, 285)	Fully connected layer
Dropout1	(*B*, 1, 285)	Regularization
Lambda	(*B, S, E*)	Reshape for attention
MHSA	(*B, S, E*)	Multi-head self-attention
LayerNorm	(*B, S, E*)	Normalize attention output
Add	(*B, S, E*)	Residual connection
Dense2 (FFN)	(*B*, 512)	Feed-forward layer
Dropout2	(*B*, 512)	Regularization
Dense3 (Out)	(*B*, 1, 285)	Final classification
Softmax	(*B*, 1, 285)	Class probabilities

#### 3.5.6 LeafSightX: proposed hybrid deep feature fusion Dense201 and InceptionV3

**Figure 5:**
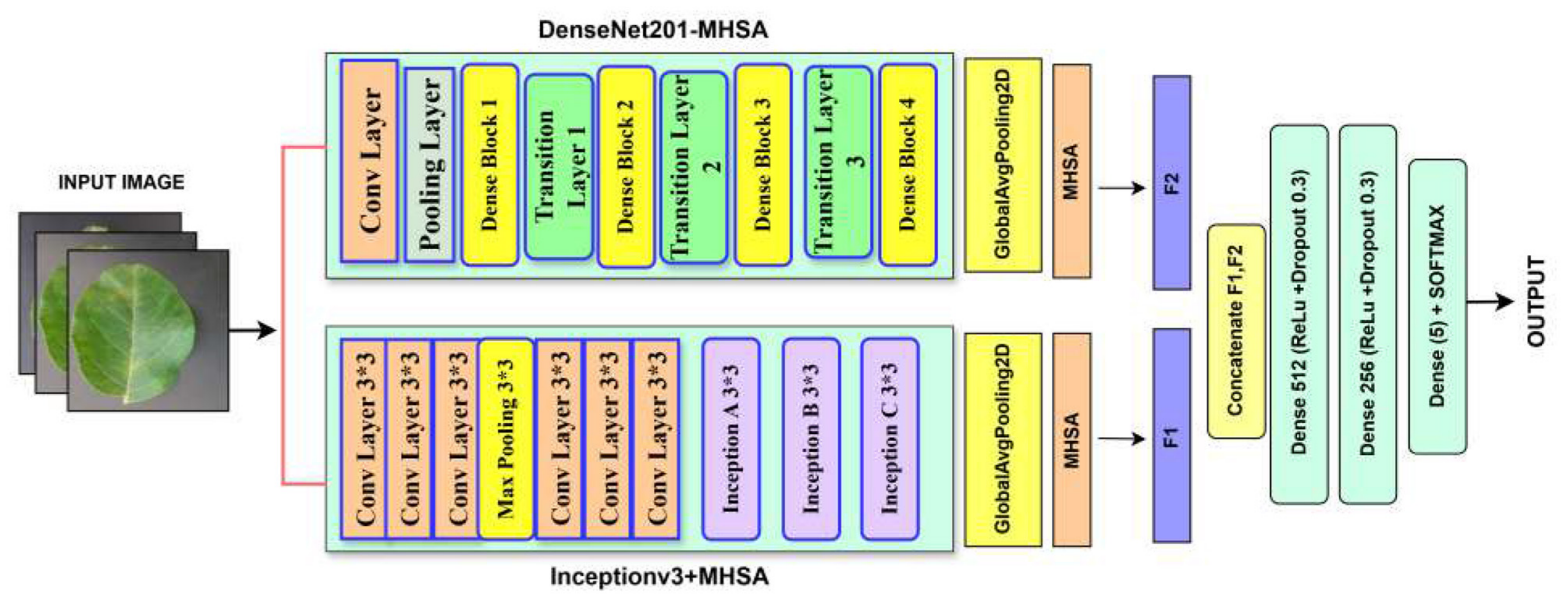
Architecture of the proposed LeafSightX model (the dual backbone feature extraction, multi-head self-attention modules, and the fusion mechanism that jointly learn discriminative representations for apple leaf disease classification).

The proposed LeafSightX model is a combination of the DenseNet201 and InceptionV3 architectures, applied to a deep feature fusion strategy complemented by Multi-Head Self-Attention structures (Li et al., 2023). The model takes in processed input images. In parallel, the two pre-trained networks each have a global average pooling and an attention layer to extract rich (and complementary) features. The versions of these features will then be concatenated and run through fully connected layers with dropout as regularization, and, lastly, classification will be performed. The architecture has a total of 114 concatenation layers, 294 convolutional layers, 295 batch normalization and activation layers each, and two Multi-Head Attention modules. Other elements include pooling layers, layer normalization, and residual connections introduced through Lambda and zero-padding layers, making the model's design tightly structured and capable of fulfilling its purpose. In detail, the LeafSightX model architecture is illustrated in Figure 5, which features a dual backbone feature extractor, self-attention modules with shared parameters across different heads, and a combination pattern that collectively learns discriminative representations for classifying apple leaf diseases. Table 11 provides a layer-wise summary of the proposed LeafSightX architecture and its training details. Algorithm 1 illustrates the overall workflow of the proposed LeafSightX model.

**Table 11:** Layer-wise summary of the LeafSightX architecture (DenseNet + InceptionV3 + MHSA).

**Layer/block**	**Output shape**	**Description**
**Input and pre-processing**		
Input layer	224 × 224 × 3	RGB input leaf image (rescaled to [0,1])
**Feature extraction branches**		
DenseNet201 backbone	7 × 7 × 1920	Pre-trained on ImageNet; all convolutional blocks frozen (no fine-tuning). Extracts dense hierarchical features with skip connections.
Global average pooling (DenseNet)	1 × 1 × 1920	Reduces spatial dimensions by averaging each feature map.
MHSA (DenseNet)	(*B*, 1920)	Multi-Head Self-Attention with 4 heads, key dimension 64; captures inter-feature dependencies.
InceptionV3 backbone	5 × 5 × 2048	Pre-trained on ImageNet; all layers frozen. Extracts multi-scale convolutional features via Inception blocks.
Global average pooling (InceptionV3)	1 × 1 × 2048	Aggregates feature maps into global feature vectors.
MHSA (InceptionV3)	(*B*, 2048)	Multi-Head Self-Attention applied to Inception features for contextual refinement.
**Fusion and classification head**		
Concatenate (feature fusion)	(*B*, 3968)	Concatenates the DenseNet201 and InceptionV3 MHSA-enhanced feature vectors.
Dense layer 1	(*B*, 512)	Fully connected layer with 512 neurons, ReLU activation, L2 regularization (1 × 10^−4^).
Dropout 1	(*B*, 512)	Dropout with 0.3 rate to prevent overfitting.
Dense layer 2	(*B*, 256)	Fully connected layer with 256 neurons, ReLU activation, L2 regularization.
Dropout 2	(*B*, 256)	Dropout with 0.3 rate.
Output layer (Softmax)	(*B*, 5)	Dense layer with 5 units (number of classes), Softmax activation to output class probabilities.
**Training and optimization details**		
Optimizer	–	Adam optimizer (learning rate adjusted via ReduceLROnPlateau).
Loss function	–	Categorical cross-entropy.
Callbacks	–	Early stopping (patience = 5), Learning rate reduction (factor = 0.5).

Algorithm 1 LeafSightX: dual-backbone transfer learning with multi-head self-attention. 
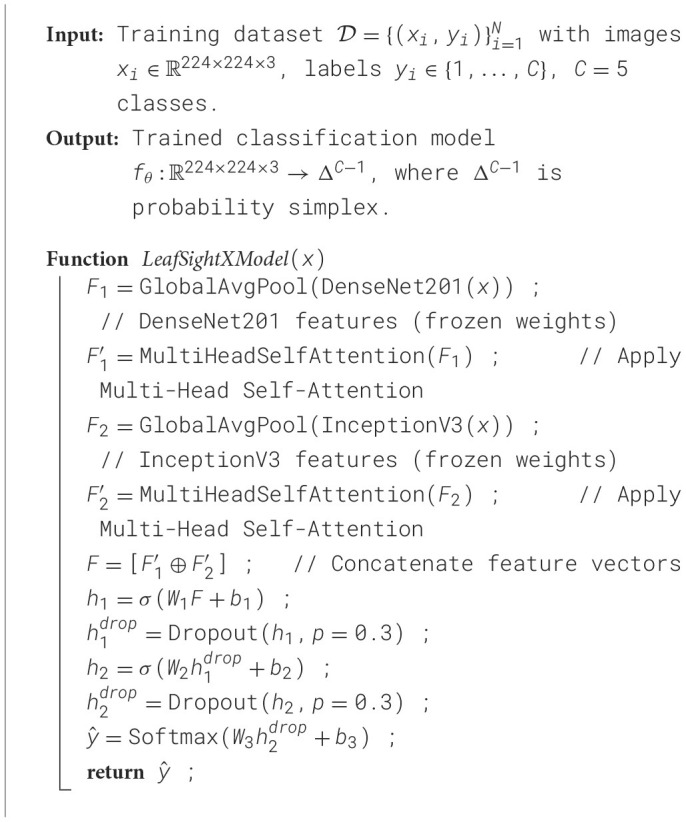

### 3.6 Model training settings

CNN baseline models and the proposed LeafSightX framework were trained with reasonable hyperparameters and approaches to provide a fair and comparable assessment. Images were resized and normalized, and pretrained weights were used to speed up convergence. Four attention heads, Multi-Head Self-Attention modules, were combined to capture global dependencies. The Adam optimizer with categorical cross-entropy loss was used to train the given model, along with regularization methods, dropout, and L2 weight decay, to prevent overfitting. The use of early stopping and learning rate scheduling addressed training robustness. In particular, it has used the ReduceLROnPlateau callback to monitor the validation loss and reduce the learning rate by a factor of 0.5 after 3 consecutive poor epochs. The overall training settings used across all models are described in Table 12.

**Table 12:** Training settings and hyperparameters for CNNs and LeafSightX.

**Parameter**	**Value/description**
Input image size	224 × 224 × 3
Pretrained models	DenseNet201, InceptionV3, VGG19, NASNetMobile, Xception
Proposed framework	LeafSightX (DenseNet201-MHSA + InceptionV3-MHSA fusion)
Base model weights	ImageNet
Attention mechanism	Multi-Head (4 heads, key_dim = 64)
Dropout rate	0.3
L2 regularization	1 × 10^−4^
Output activation	Softmax (multi-class)
Loss function	Categorical crossentropy
Optimizer	Adam
Learning rate	1 × 10^−3^ (default)
Batch size	32
Epochs	Up to 40 (with early stopping)
Early stopping	Monitor val_loss, patience = 5
ReduceLROnPlateau	Monitor val_loss, factor = 0.5, patience = 3
Shuffle data	True (train), False (val/test)
Color mode	RGB
Number of classes	5 (Healthy, Alt., Rust, Gray, Brown Spot)

### 3.7 Proposed model explainability

To enhance the interpretability of the LeafSightX model, we employed Grad-CAM, which operates on both DenseNet201 and InceptionV3 backbones. Grad-CAM provides class-discriminative heatmaps by calculating the gradient of a class score over the feature maps of the preceding convolutional layer. The approach can help identify areas in the input image that make the most significant contribution to the model's predictions.

In every backbone, activations of the last convolutional layer were used to generate heatmaps of the original images of leaves. These visual explanations showed that DenseNet201 and InceptionV3 both focused on biologically significant areas, including disease lesions, texture alterations, and color aberrations. Grad-CAM demonstrates that the LeafSightX model determines actions by considering relevant visual elements, thereby increasing transparency and reliability in disease classification.

### 3.8 Evaluation metrics

To comprehensively assess the performance of the proposed LeafSightX model, multiple evaluation metrics were employed.

**Accuracy** measures the overall correctness of the predictions and is defined as:


$$\text{Accuracy}=\frac{TP+TN}{TP+TN+FP+FN}$$

where *TP* is true positives, *TN* is true negatives, *FP* is false positives, and *FN* is false negatives.

**Precision** evaluates how well the model identifies positive cases, defined as:


$$\text{Precision}=\frac{TP}{TP+FP}$$

**Recall** measures the model's ability to detect actual positive cases:


$$\text{Recall}=\frac{TP}{TP+FN}$$

**F1-Score** balances precision and recall using their harmonic mean:


$$F1=2\cdot \frac{\text{Precision}\cdot \text{Recall}}{\text{Precision}+\text{Recall}}$$

A **confusion matrix** summarizes all *TP*, *FP*, *TN*, and *FN* values, providing a clear view of classification errors.

**Area Under the ROC Curve (AUC)** measures the model's ability to distinguish between classes across different thresholds. In contrast, **Precision-Recall AUC (PR AUC)** quantifies the trade-off between precision and recall over varying thresholds.

Finally, the robustness and reliability of LeafSightX were evaluated against baseline models using statistical significance testing, ensuring that observed improvements are not due to chance.

## 4 Results and discussion

In this section, we systematically analyze the performance of the proposed *LeafSightX* model in comparison to established baselines. We explore its accuracy, robustness, and interpretability through extensive experiments, emphasizing its potential to advance automated plant disease diagnosis. The discussion highlights key insights gained from the results and situates our findings within the broader context of agricultural AI applications.

### 4.1 Model performance overview

The classification accuracy of all the evaluated models was comparatively assessed on the training, validation, and test sets and is presented in Table 13. The training accuracies of all architectures were high, indicating that features can be well learned for leaf disease classification. DenseNet201 and InceptionV3 were found to generalize well, with both validation and test accuracy well above 98%, whereas VGG19 and NASNetMobile performed relatively poorly, possibly because features are not extracted optimally, or the models are too small. The proposed LeafSightX model outperformed all baselines, achieving 99.02% and 99.64% accuracy on the validation and test sets, respectively, which are similar to its training accuracy of 99.98%. The slight performance difference reflects the excellent overfitting mitigation enabled by the dual-backbone fusion and MHSA modules, which enhance discriminative feature representation. These findings highlight the strengths and potential of LeafSightX for real-world applications as an agricultural diagnostic system.

**Table 13:** Comparison of train, validation, and test accuracy for different models.

**Model**	**Train accuracy**	**Validation accuracy**	**Test accuracy**
DenseNet201	0.9980	0.9890	0.9878
InceptionV3	0.9989	0.9841	0.9830
VGG19	0.9930	0.9670	0.9732
Xception	0.9988	0.9792	0.9781
NASNetMobile	0.9819	0.9609	0.9611
LeafSightX (proposed)	0.9998	0.9902	0.9964

### 4.2 Comprehensive performance metrics: precision, recall, F1-score, AUC, and PR AUC

To provide a comprehensive evaluation beyond accuracy, Table 14 presents additional performance metrics including precision, recall, F1 score, AUC, and PR AUC across training, validation, and test sets. These metrics assess class balance, model sensitivity, and confidence calibration. The proposed LeafSightX model consistently outperforms all baseline models, achieving mean precision, recall, and F1 scores of more than 0.99 on both the validation and test sets. The high recall with low false positives implies strong identification of diseased cases, whereas F1 scores indicate a balanced classification. AUC and PR AUC values near 1.000 indicate high discrimination and a strong precision-recall trade-off, which is significant when the classes are unbalanced. Conversely, there is a visible drop in these measures between the training, validation, and test sets when using baseline models, indicating overfitting and poor generalization. These findings support the robustness, reliability, and applicability of the proposed model for the accurate diagnosis of leaf diseases in precision agriculture.

**Table 14:** Average precision, recall, F1-score, AUC, and PR-AUC for train, validation, and test sets.

**Model**	**Dataset**	**Precision**	**Recall**	**F1-score**	**AUC**	**PR AUC**
DenseNet201	Train	0.9982	0.9982	0.9982	1.0000	1.0000
	Validation	0.9903	0.9891	0.9897	0.9998	0.9995
	Test	0.9898	0.9876	0.9887	0.9996	0.9988
InceptionV3	Train	0.9991	0.9991	0.9991	1.0000	1.0000
	Validation	0.9912	0.9866	0.9889	0.9997	0.9991
	Test	0.9920	0.9838	0.9879	0.9995	0.9986
VGG19	Train	0.9940	0.9940	0.9940	0.9999	0.9997
	Validation	0.9737	0.9679	0.9708	0.9991	0.9972
	Test	0.9796	0.9709	0.9752	0.9987	0.9962
Xception	Train	0.9990	0.9988	0.9989	1.0000	1.0000
	Validation	0.9886	0.9814	0.9850	0.9992	0.9975
	Test	0.9861	0.9803	0.9832	0.9991	0.9974
NASNetMobile	Train	0.9832	0.9799	0.9815	0.9997	0.9991
	Validation	0.9735	0.9601	0.9668	0.9986	0.9959
	Test	0.9688	0.9392	0.9538	0.9981	0.9946
LeafSightX (proposed)	Train	0.9999	0.9997	0.9998	1.0000	1.0000
	Validation	0.9957	0.9913	0.9935	0.9998	0.9994
	Test	0.9964	0.9960	0.9962	1.0000	1.0000

### 4.3 Assessing model generalization via 5-fold cross-validation

To assess the models' strength and generalization, a 5-fold cross-validation was conducted, and the results are shown in Table 15. In 5-fold cross-validation, the dataset is divided into five equal parts. Each part is used once as a validation set, while the remaining four are used for training. The process repeats five times to ensure all data is tested. LeafSightX achieved the best performance across all folds, with an average validation accuracy of 99.19% and a low standard deviation of 0.14%, indicating stable performance across data splits. Its average Cohen's Kappa of 0.9917 validates predictions well beyond chance. Validation AUC and PR AUC were also very high, 0.9998 and 0.9994, respectively, indicating good discriminative power and multi-class performance even when classes are unbalanced. The nearest baseline was DenseNet201, achieving an average validation accuracy of 99.02% and a Kappa of 0.9876. InceptionV3 and Xception obtained fairly good results of about 97%–98% accuracy, but with a bit more variation. VGG19 and NASNetMobile showed lower accuracy and Kappa scores, and poorer generalization, likely due to fewer features. On the whole, these findings indicate that LeafSightX exhibits consistent, reliable, and robust performance and justify the efficiency of dual-backbone fusion and MHSA for managing leaf diseases in practice.

**Table 15:** 5-Fold validation metrics for different models.

**Model**	**Fold**	**Val accuracy**	**Val Kappa**	**Val AUC**	**Val PR AUC**
DenseNet201	1	0.9927	0.9907	0.9999	0.9996
	2	0.9902	0.9876	0.9999	0.9997
	3	0.9817	0.9768	0.9999	0.9996
	4	0.9902	0.9876	0.9999	0.9997
	5	0.9963	0.9954	0.9999	0.9998
	Avg ± SD	0.9902 ± 0.0051	0.9876 ± 0.0063	0.9999 ± 0.0	0.9997 ± 0.0001
InceptionV3	1	0.9810	0.9759	0.9985	0.9956
	2	0.9703	0.9625	0.9988	0.9963
	3	0.9779	0.9720	0.9995	0.9983
	4	0.9741	0.9672	0.9987	0.9963
	5	0.9718	0.9641	0.9984	0.9947
	Avg ± SD	0.9750 ± 0.0046	0.9683 ± 0.0048	0.9988 ± 0.0004	0.9962 ± 0.0012
VGG19	1	0.9703	0.9624	0.9987	0.9964
	2	0.9650	0.9558	0.9987	0.9960
	3	0.9787	0.9730	0.9990	0.9969
	4	0.9596	0.9488	0.9978	0.9931
	5	0.9649	0.9553	0.9986	0.9956
	Avg ± SD	0.9677 ± 0.0067	0.9591 ± 0.0077	0.9986 ± 0.0004	0.9956 ± 0.0013
Xception	1	0.9703	0.9624	0.9989	0.9967
	2	0.9673	0.9587	0.9982	0.9943
	3	0.9832	0.9788	0.9991	0.9977
	4	0.9787	0.9730	0.9993	0.9976
	5	0.9657	0.9562	0.9989	0.9969
	Avg ± SD	0.9730 ± 0.0067	0.9658 ± 0.0078	0.9989 ± 0.0004	0.9966 ± 0.0013
NASNetMobile	1	0.9612	0.9509	0.9979	0.9933
	2	0.9756	0.9692	0.9988	0.9967
	3	0.9764	0.9701	0.9990	0.9973
	4	0.9642	0.9547	0.9983	0.9941
	5	0.9611	0.9505	0.9978	0.9933
	Avg ± SD	0.9677 ± 0.0074	0.9591 ± 0.0083	0.9984 ± 0.0005	0.9949 ± 0.0017
LeafSightx	1	0.9924	0.9904	0.9999	0.9997
	2	0.9943	0.9928	0.9997	0.9992
	3	0.9899	0.9871	0.9998	0.9991
	4	0.9911	0.9888	0.9999	0.9997
	5	0.9918	0.9896	0.9998	0.9993
	Avg ± SD	0.9919 ± 0.0014	0.9917 ± 0.0020	0.9998 ± 0.0001	0.9994 ± 0.0003

### 4.4 Computational cost analysis

**Figure 6:**
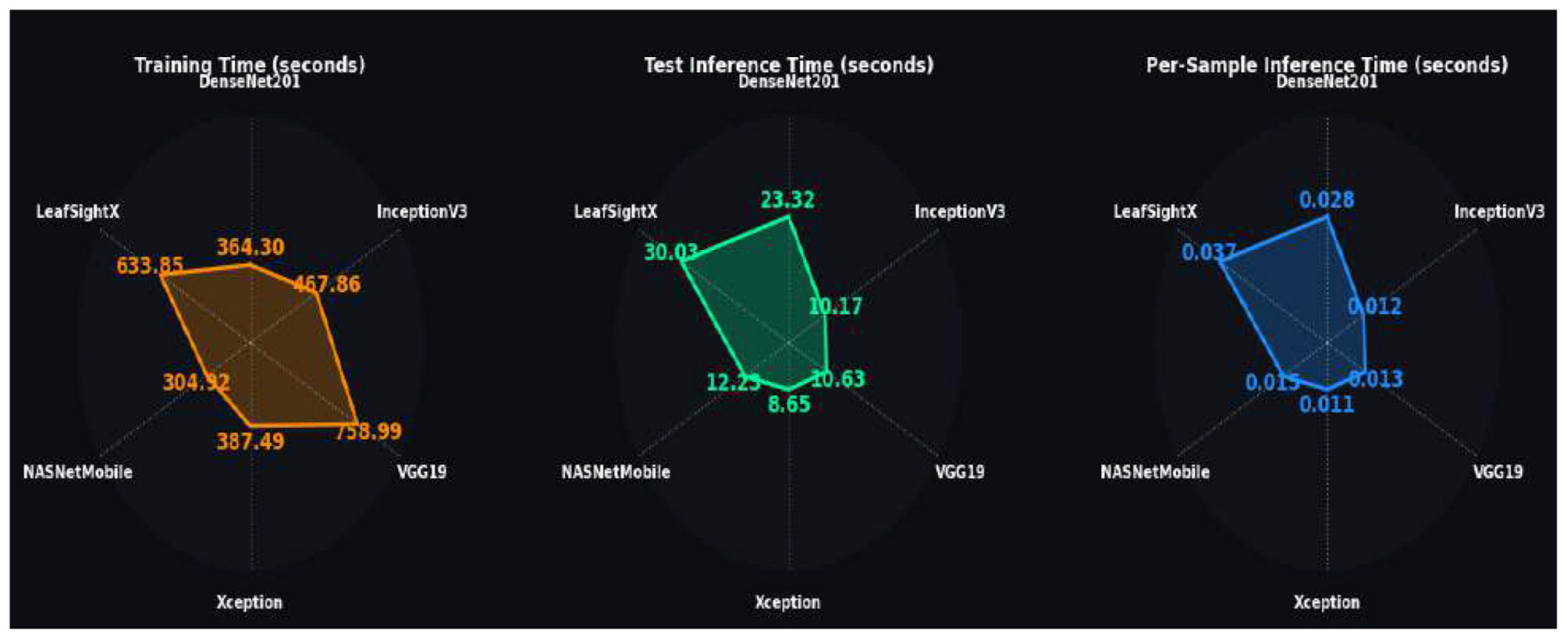
Radar plot illustrating the computational cost of six deep learning models for Apple Leaf disease classification, including training time, test inference time, and per-sample inference time.

The radar chart in Figure 6 illustrates the computational cost of six deep learning models used for Apple Leaf disease classification in terms of training time, test inference time, and per-sample inference time. On the whole, DenseNet201 has a moderate training time of 364.30 seconds, a relatively high test inference time of 23.32 seconds, and a per-sample time of 0.0284 seconds, compared to InceptionV3, which has the longest training time of 467.86 seconds, the highest test inference time of 10.17 seconds, and a per-sample time of 0.0124 seconds, respectively. In comparison, the VGG19 has a better training time of 758.99 seconds, with test inference times of 10.63 seconds and 0.0129 seconds per sample, respectively. Conversely, Xception proves efficient across the board, with training, test, and per-sample inference times of 387.49, 8.65, and 0.0105 seconds, respectively. Equally, NASNetMobile has the lowest training time of 304.92 seconds, with average test inference and per-sample times of 12.23 seconds and 0.0149 seconds, respectively. Lastly, the proposed LeafSightX model has comparatively high computational costs: 633.85 seconds for training, 30.03 seconds for test inference, and 0.0365 seconds per-sample inference. Thus, the chart can serve well to indicate the trade-offs in the training and inference efficiency of the models for Apple Leaf disease detection.

### 4.5 Performance evaluation via learning curves, confusion matrix, and AUC-based metrics

To comprehensively assess the proposed model's performance, we employ a suite of visual evaluation tools. These include learning curves to monitor training dynamics, a confusion matrix to analyze class-wise predictions, and AUC-based metrics to evaluate classification quality under varying thresholds. Such visualizations provide deeper insight into the model's strengths, weaknesses, and generalization behavior.

#### 4.5.1 Performance trends across training epochs

**Figure 7:**
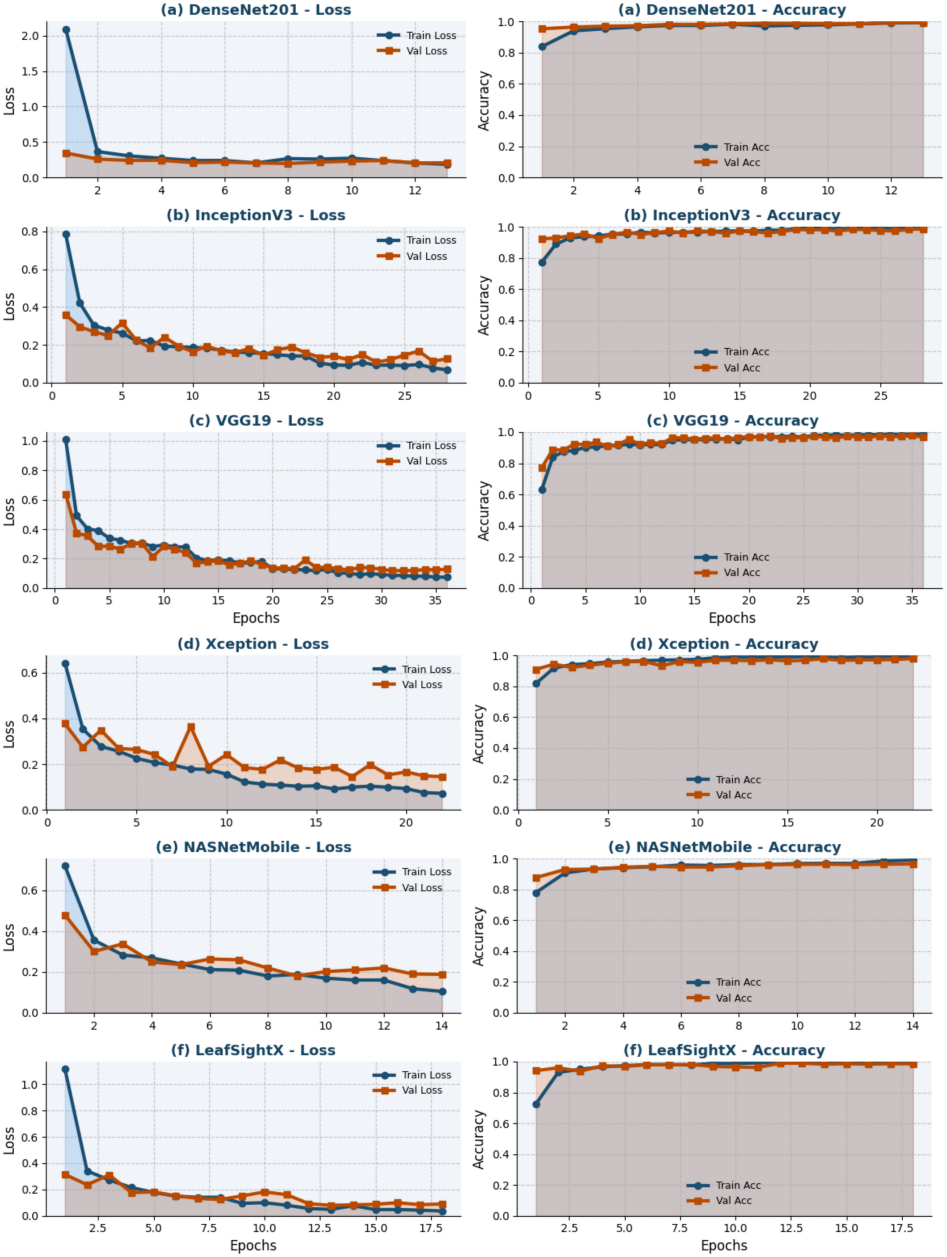
Training and validation loss and accuracy curves across epochs for six models: **(a)** DenseNet201, **(b)** InceptionV3, **(c)** VGG19, **(d)** Xception, **(e)** NASNetMobile, and **(f)** LeafSightX. The figure provides a comprehensive comparison of training and validation performance across all models.

Figure 7 shows the training and validation loss curves of all evaluated models. To start with, the loss curves for DenseNet201 demonstrate effective learning, with training and validation losses steadily declining from initial values of 2.0927 and 0.3448, respectively, to 0.2059, suggesting robust generalization with little overfitting. Concurrently, InceptionV3 shows a slight decrease in training loss [0.7851 to 0.0680], but validation loss ranges from 0.3592 to 0.1259, suggesting slight fluctuations but good generalization. Conversely, VGG19 converges more slowly once the training loss has reduced to 0.0734 and validation loss to 0.1281, indicating higher computational and slower optimization. Conversely, Xception shows a more fluent and efficient convergence: the training loss is reduced to 0.0718, and the validation loss to 0.1450. NASNetMobile shows rapid convergence, with training and validation losses dropping to 0.1048 and 0.1881, respectively, suggesting a fast learning process and sensible levels of generalization. Lastly, the LeafSightX model achieves the best performance: the training loss drops to 0.0365, and the validation loss to 0.0900, demonstrating excellent optimization, stability, and generalization. Overall, the loss curves indicate that all models converge, and LeafSightX has the most efficient and effective training dynamics among all other evaluated models.

#### 4.5.2 Confusion matrix analysis

**Figure 8:**
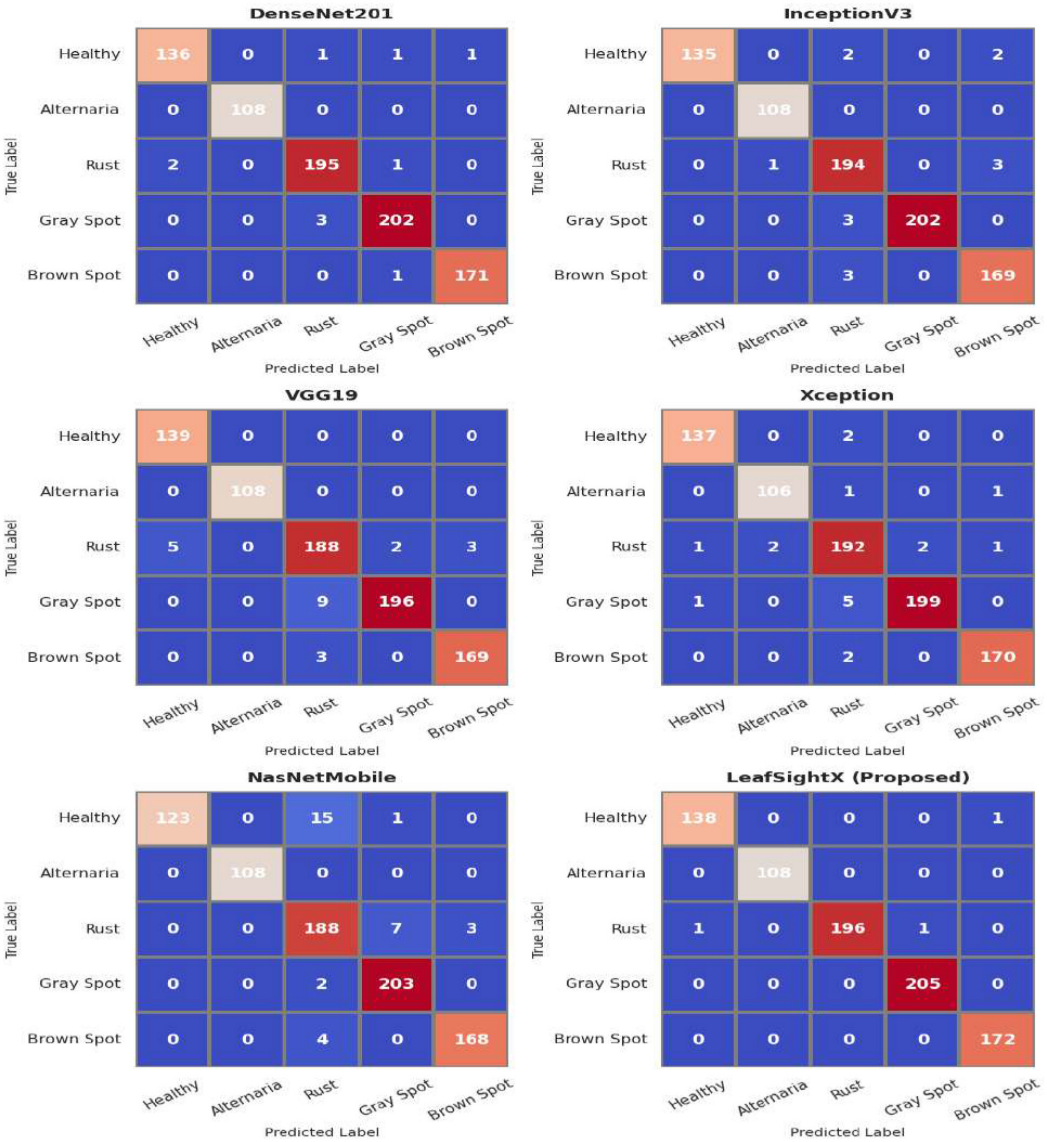
Confusion matrices for various deep learning models on apple leaf disease classification, showing true vs. predicted labels for five classes: Healthy leaf, Alternaria leaf spot, Rust, Gray spot, and Brown spot.

The classification performances of the models in classifying leaf diseases are shown in the confusion matrices in Figure 8. DenseNet201 accurately recognized the majority of classes, including 136 healthy leaves, all cases of Alternaria leaf spot, and rust and gray spot, which were slightly confused with brown spot. The same was observed in InceptionV3, with the minor misclassifications being between rust and brown spot, but healthy leaves and Alternaria leaf spot were generally correct. VGG19 had more misclassifications, particularly on rust, gray spot, and healthy leaves, suggesting a low discriminative capacity. Xception produced more evenly distributed errors, with most samples correctly classified and only slight misclassifications in a few categories. NASNetMobile was more confused, especially with healthy leaves being classified as rust 15 times, 7 Rust predicted as Gray Spot, 4 Brows spot predicted as Rust, but the rest of the classes had few misclassifications. The proposed model, LeafSightX, was the most effective, with the fewest misclassifications. There were 138 correct classifications of healthy leaves and one incorrect classification of brown spot. Alternaria leaf spot contained no errors. Rust was correct on 196 of its predictions with only two small misclassifications. In all 205 and 172 cases, respectively, the gray and brown spots were correctly identified. The findings indicate that LeafSightX is more accurate and less confusing, especially in rust, gray spot, and brown spot. The findings affirm the conclusion that LeafSightX performs better in the performance-based class, especially against rust, gray spot, and brown spot, indicating superior performance compared to the other models.

### 4.6 Model calibration, statistical significance, and reliability metrics

Additional complementary indicators and tests were implemented to conduct a comprehensive assessment of the reliability, calibration, and statistical significance of the measured classification models. The Brier Score is a metric that assesses the precision of probabilistic forecasts and their calibration across all classes. The nonparametric *p*-values were robust, and permutation testing was used to determine whether the observed best accuracies are substantially larger than those expected by chance. To establish a competition on confidence levels for accuracy measurements, bootstrap resampling was implemented to provide insight into the performance's stability. Lastly, Cohen's Kappa statistic measures the extent to which predicted labels match the actual ones above chance level, thereby serving as an additional metric of reliability. The average Brier Scores, accuracy, permutation test *p*-values, bootstrap accuracy with 95% confidence interval, and Cohen's Kappa scores are summarized in Table 16. These statistics are calculated for all models.

**Table 16:** Average brier scores, accuracy, permutation *p*-values, bootstrap accuracy (95% CI), and Cohen's kappa.

**Model**	**Dataset**	**Avg. brier score**	**Accuracy**	**Permutation**	**Bootstrap accuracy**	**95% CI**	**Kappa (test)**
				***p*** **-value**			
DenseNet201	Train	0.0006	0.9980	0.0001	0.9980	0.9968–0.9989	–
	Validation	0.0035	0.9890	0.0001	0.9890	0.9817–0.9951	–
	Test	0.0039	0.9878	0.0001	0.9878	0.9805–0.9939	0.9846
InceptionV3	Train	0.0004	0.9989	0.0001	0.9989	0.9982–0.9997	–
	Validation	0.0048	0.9841	0.0001	0.9843	0.9744–0.9927	–
	Test	0.0049	0.9830	0.0001	0.9826	0.9732–0.9915	0.9784
VGG19	Train	0.0023	0.9930	0.0009	0.9930	0.9910–0.9948	–
	Validation	0.0090	0.9670	0.0009	0.9671	0.9536–0.9792	–
	Test	0.0085	0.9732	0.0009	0.9733	0.9611–0.9842	0.9661
Xception	Train	0.0005	0.9988	0.0009	0.9988	0.9980–0.9995	–
	Validation	0.0073	0.9792	0.0009	0.9792	0.9683–0.9890	–
	Test	0.0065	0.9781	0.0009	0.9777	0.9672–0.9866	0.9723
NASNetMobile	Train	0.0051	0.9819	0.0009	0.9819	0.9787–0.9849	–
	Validation	0.0110	0.9609	0.0009	0.9609	0.9487–0.9744	–
	Test	0.0122	0.9611	0.0009	0.9611	0.9477–0.9732	0.9506
LeafSightX (Proposed)	Train	0.0001	0.9998	0.0009	0.9999	0.9995–1.0000	–
	Validation	0.0030	0.9902	0.0009	0.9902	0.9829–0.9963	–
	Test	0.0011	0.9964	0.0009	0.9963	0.9915–1.0000	0.9954

The low average Brier Scores across all models, ranging from 0.0001 for the proposed LeafSightX model on the training set to 0.0122 for NASNetMobile on the test set, indicate perfect calibration of the predicted probabilities and well-calibrated confidence estimates. Accuracy scores also emphasize the models' predictive abilities: LeafSightX achieves 99.98 on the training data and 99.64 on the test data, whereas all models achieve over 96 on the test data. Its results are confirmed by p-values from the permutation tests, which are always less than 0.001, indicating that the classification performance significantly outperforms random chance alone. The 95% confidence intervals obtained with the Bootstrap are stringent; i.e., the accuracy of the LeafSightX test is estimated at 99.15–100.00, and the accuracy of the DenseNet201 test is estimated at 98.05–99.39, indicating a stable and accurate range of estimates. Additionally, the Cohen Kappa values in the test set are impressive (0.9506, NASSNetMobile; 0.9954, LeafSightX), demonstrating the reliability of the models' labels despite the predicted and actual labels achieving near-perfect performance, which is significantly better than chance.

**Figure 9:**
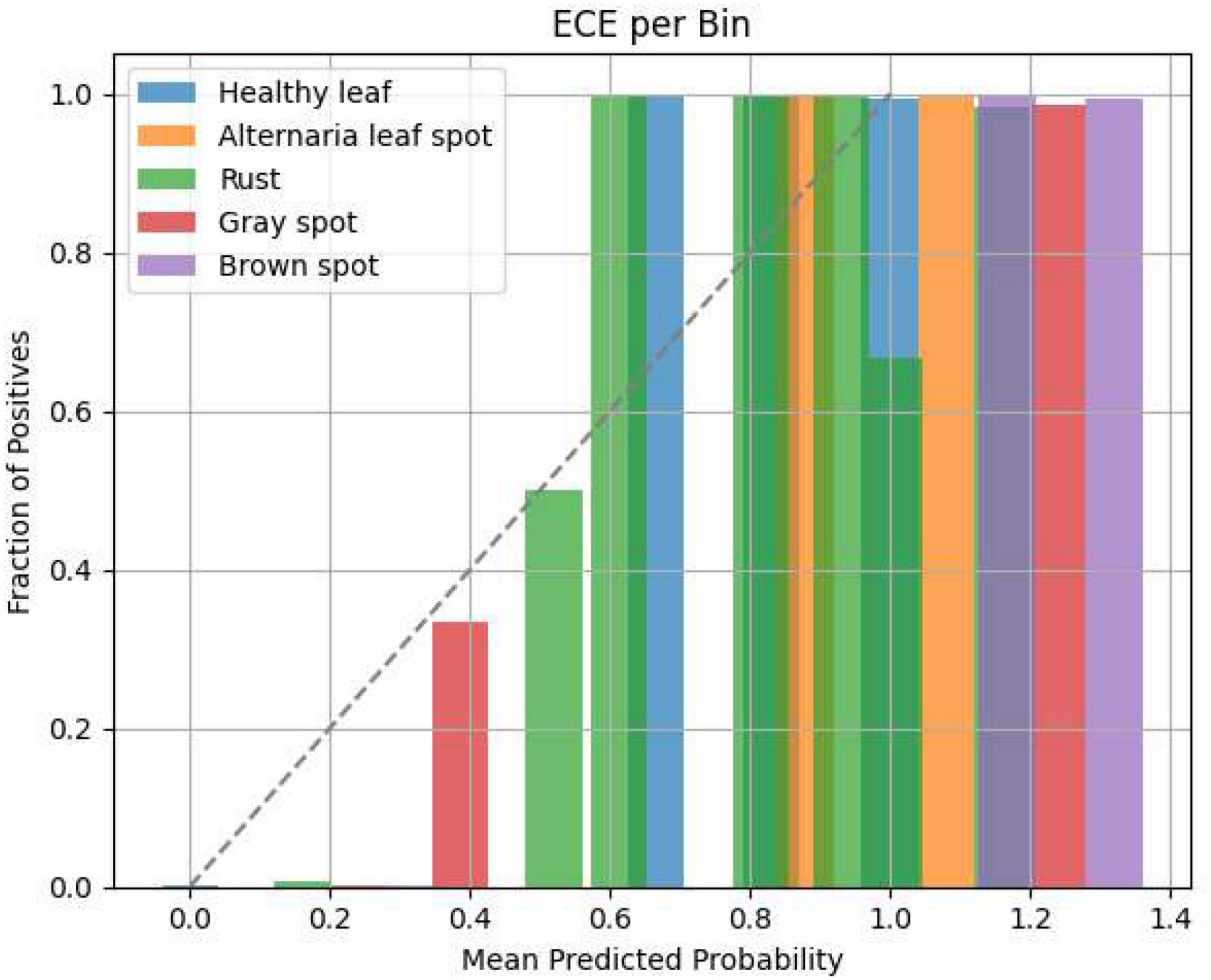
Expected calibration error (ECE) bar plot for the proposed LeafSightX model, showing the alignment between predicted probabilities and actual outcomes across different confidence bins.

All of these complementary measures, granted by probabilistic calibration using the Brier Score, statistical verification using permutation testing, quantification of uncertainty using bootstrap confidence intervals, and agreement and consistency using the Cohen Kappa, form an in-depth and stringent evaluation scheme. The method has been used to create transparency and reliability in model evaluation, and this strategy has supported the real-world applicability of such classifiers for disease identification in agriculture. To evaluate the calibration of our proposed LeafSightX model, we present the Expected Calibration Error (ECE) bar plot, which illustrates how well the predicted probabilities align with actual outcomes across different confidence bins, as presented in Figure 9.

Figure 9 presents the Expected Calibration Error (ECE) per bin of five classes of plant leaf disease. The x-axis is the average predicted probability, and the y-axis is the proportion of positives in each probability bin. The color-coded bars indicate the distribution of predicted probabilities of each disease group. The dotted diagonal line indicates that the calibration is perfect; that is, the predicted probabilities are equal to the actual class frequencies. As shown in the figure, most classes are well-calibrated in terms of the predictability of their probabilities, with their bars tending to closely or broadly follow the diagonal line. Nevertheless, Gray spot and Brown spot exhibit calibration flaws, especially in higher-probability bins, suggesting the model is either overconfident or underconfident for these classes. We also include the Confidence Distribution Curve and Max-confidence Histogram for the proposed LeafSightX system, to further assess the model's confidence across all disease classes. These additions provide more information about the model's behavior and, as such, help evaluate and optimize model calibration, especially for more challenging courses such as Gray spot and Brown spot.

**Figure 10:**
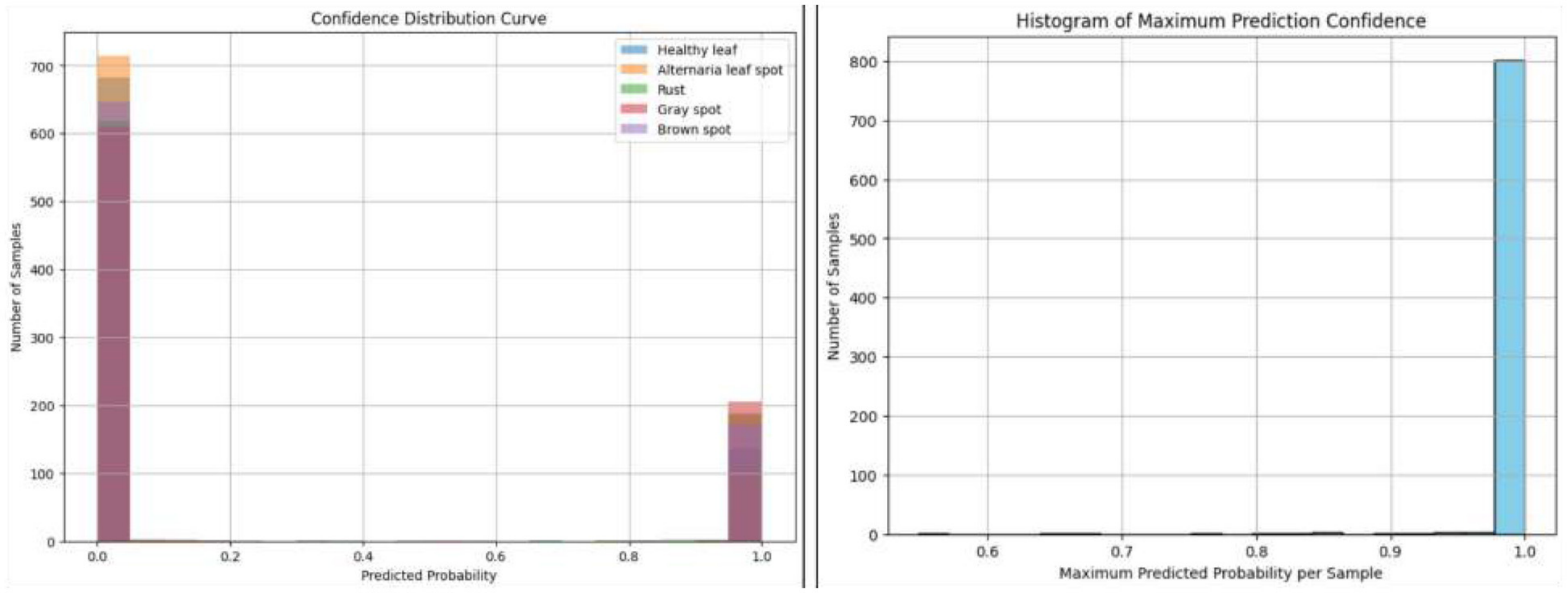
Confidence distribution curve and maximum prediction confidence histogram for the proposed LeafSightX system.

Figure 10 shows the Confidence Distribution Curve (left) and the Histogram of Maximum Prediction Confidence (right) for the proposed LeafSightX system. The Confidence Distribution Curve shows that many predictions have a distinctly strong score of zero or one, indicating that the model is very sure in its predictions. In contrast, only a small number have a moderate score. This suggests that the model is likely to give firm predictions, either sure of its classification or unsure, instead of giving a wide spectrum of probability values.

This is further supported by the Histogram of Maximum Prediction Confidence, which indicates that most samples have maximum predicted probabilities close to 1. It means the model is always certain about its predictions, though the number of samples with lower confidence is significantly lower. The results imply that the model is confident in its predictions, but additional calibration may be needed to better handle less certain cases.

### 4.7 Ablation study: evaluating the impact of multi-head self attention on LeafSightX and backbone models

In our ablation study, we evaluated the test accuracy of DenseNet201, InceptionV3, and their combinations without Multi-Head Self Attention (MHSA). Without MHSA, DenseNet201, InceptionV3, and the DenseNet + InceptionV3 fusion achieved test accuracies of 0.9854, 0.9672, and 0.9927, respectively, as observed from Table 1. After integrating MHSA, DenseNet201, and InceptionV3, the models achieved 0.9878 and 0.9830, while the fusion model reached the highest accuracy of 0.9964. These results indicate that MHSA improves the model's ability to focus on relevant spatial features, enhancing classification performance.

The combination of DenseNet201 and InceptionV3 with MHSA outperforms all individual models. The improvement over the fusion model without MHSA highlights the importance of attention mechanisms in identifying key patterns and producing more reliable predictions. These findings confirm LeafSightX's effectiveness in achieving superior accuracy and generalization, establishing it as a robust tool for leaf disease classification and agricultural diagnostics.

### 4.8 Interpretation of learned features in the fusion backbone using Grad-CAM

**Figure 11:**
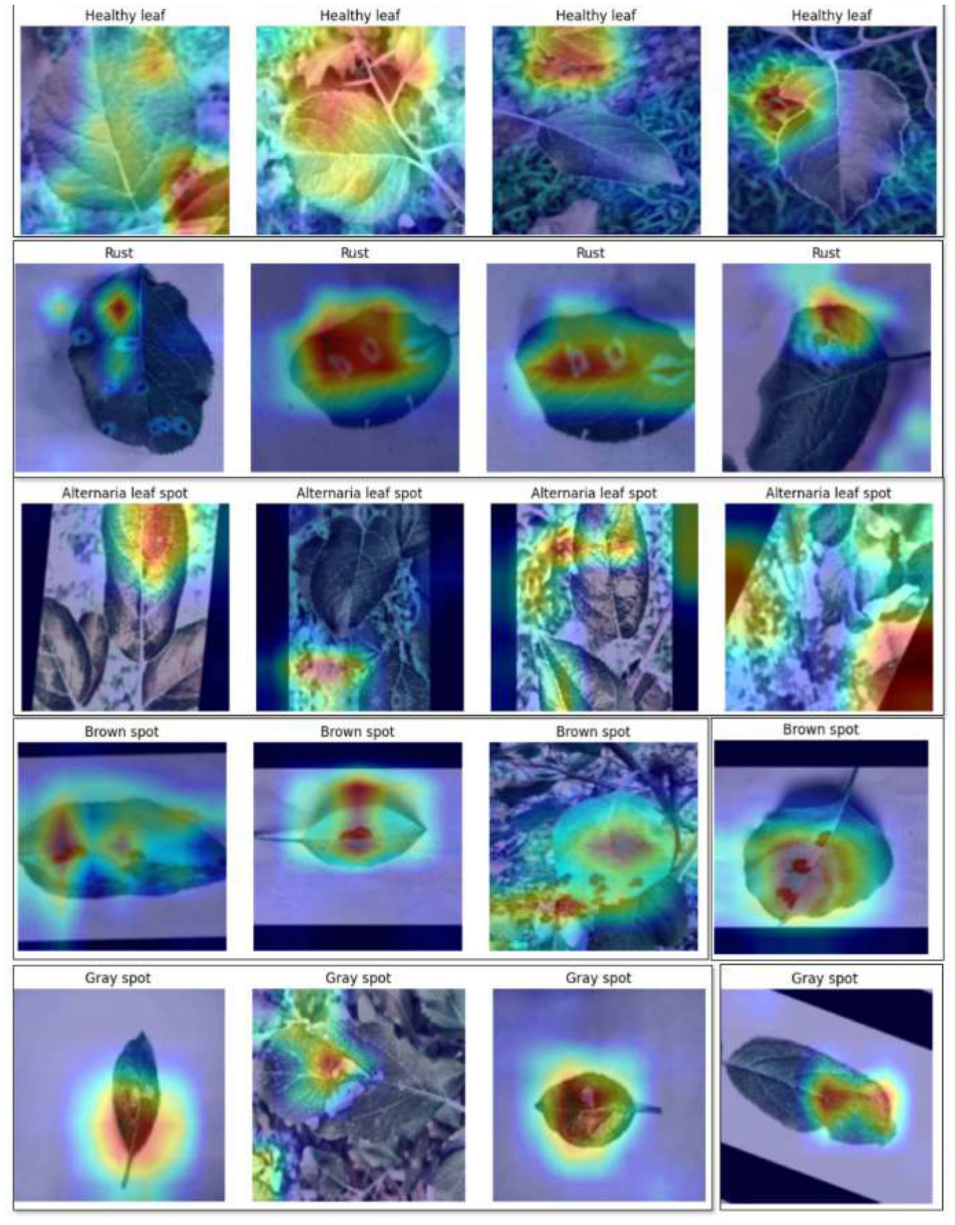
Grad-CAM visualization for DenseNet201 backbone.

**Figure 12:**
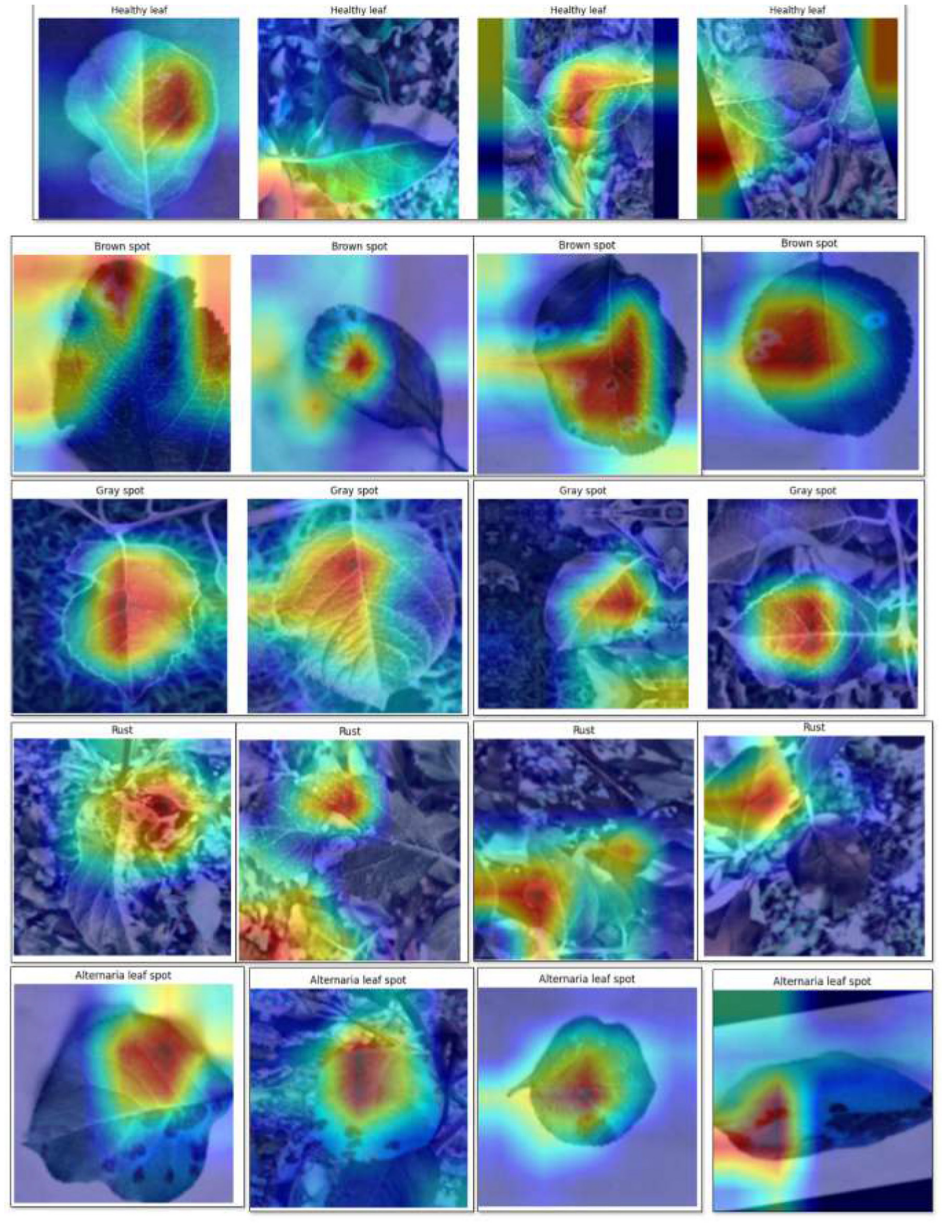
Grad-CAM visualization for InceptionV3 backbone.

The Grad-CAM visualizations for the DenseNet201 and InceptionV3 backbones are shown in Figures 11, 12, respectively, highlighting the spatial regions of leaf images that most influence model predictions. The Grad-CAM visualizations of DenseNet201 and InceptionV3 as backbone models are presented in Figures 11, 12, respectively. These backbones are the main feature extractors used in LeafSightX, and the heatmaps provide insight into their pattern preferences for identifying disease-specific features before fusion, which is helpful for interpretation and reliability.

Figure 11 indicates that DenseNet201 concentrates on the regions of the affected places that include water-soaked blotches, necrotic lesions, and distortion of the edges of the leaf. MHSA is yet another refinement of this focus, making the localization features more semantically precise and relevant. These findings indicate that DenseNet201 can capture physiologically significant patterns, consistent with visual clues identified by plant pathologists, and provide valuable additions to disease classification.

Similarly, in Figure 12, the Grad-CAM heatmaps of the InceptionV3 backbone highlight critical indicators, including textural changes, fungal plaques, and tissue atrophy. These visualizations show that InceptionV3 effectively identifies disease-relevant regions, capturing precise patterns essential for accurate diagnosis. The backbone demonstrates the ability to focus on disease-specific variations across spatial scales, enhancing interpretability and reliability. Analyzing individual backbones is crucial for understanding and validating the model's decision-making, especially in domains like agriculture and plant pathology.

Though Grad-CAM can be successfully used to visualize the spatial attention of individual backbones, feature fusion, which is repeated concatenation, then attentions and fully connected layers, breaks the spatial alignment of features to the original image, and reliable Grad-CAM heatmaps of the fused output are technically infeasible. However, Grad-CAM visualizations of the individual backbones are highly interpretable. Regions of interest are always aligned with areas affected by the disease and verified as biologically relevant by plant pathology experts, so it is plausible to expect that the fused LeafSightX model will capture these important features even better and increase its interpretability and diagnostic accuracy.

### 4.9 Comparative results using an additional dataset

Table 17 presents the comparative results of several deep learning architectures evaluated on an additional dataset (Dhar, 2023). The performance comparison shows that all models achieved consistently high accuracy across training, validation, and test sets, indicating strong generalization. Among them, the proposed LeafSightX model achieved the best overall performance, with a test accuracy of 0.9969 and an F1-score of 0.9970, surpassing all baseline architectures. DenseNet201 and InceptionV3 also demonstrated strong performance, achieving test accuracies over 0.99 and near-perfect AUC and PR AUC values. However, their marginally lower precision and recall suggest a minor imbalance in class prediction compared to the proposed method. The consistent superiority of LeafSightX across all metrics, including both AUC (0.9999) and PR AUC (1.0000), confirms its robustness and adaptability to unseen data. These results validate the scalability and generalization potential of the proposed framework, showing that it maintains high discriminative power when applied to datasets beyond the primary experimental setup.

**Table 17:** Comparative performance of deep learning models on the apple leaf disease dataset (additional dataset results).

**Model**	**Accuracy**			**Performance metrics**			**AUC scores**	
	**Train**	**Validation**	**Test**	**Precision**	**Recall**	**F1-score**	**AUC**	**PR AUC**
DenseNet201	1.0000	0.9977	0.9964	0.9964	0.9965	0.9965	0.9999	0.9999
InceptionV3	0.9999	0.9884	0.9882	0.9882	0.9883	0.9882	0.9998	0.9994
VGG19	0.9887	0.9766	0.9779	0.9779	0.9778	0.9779	0.9990	0.9973
Xception	0.9999	0.9874	0.9887	0.9888	0.9887	0.9888	0.9997	0.9992
NasNetMobile	1.0000	0.9889	0.9884	0.9886	0.9884	0.9885	0.9997	0.9991
LeafSightX (proposed)	1.0000	0.9982	0.9969	0.9970	0.9970	0.9970	0.9999	1.0000

### 4.10 Benchmarking LeafSightX against existing literature

The current deep learning models employed to predict apple leaf maladies are compared in detail with LeafSightX in Table 18. The suggested LeafSightX achieves an impressive 99.64% accuracy, surpassing all evaluated approaches, which generally report accuracies ranging from 66.8% to 99.51%. This outstanding outcome underscores the efficacy of a double-backbone architecture integrating DenseNet201 and InceptionV3 with MHSA, enhancing the model's ability to extract both local and global features of the disease from leaf images. Unlike most prior studies, LeafSightX is the inaugural work to integrate XAI, thereby ensuring transparent, interpretable predictions. In conjunction with heightened trust among end users, Grad-CAM heatmaps can help agricultural specialists derive actionable insights by highlighting diseased areas of leaves. Moreover, LeafSightX achieves these enhancements without imposing a significant computational burden, while preserving efficient runtime inference and resource allocation. The trade-off between high accuracy, interpretability, and computational intensity makes LeafSightX viable for application in a low-resource agricultural context. Consequently, our framework bridges the divide between advanced research and practical application, resulting in more user-friendly and dependable instruments for diagnosing plant diseases.

**Table 18:** Comparative analysis of existing models and *LeafSightX* for apple leaf disease detection.

**References**	**Model**	**Accuracy**	**XAI**
Liu and Li (2024)	A-Net	92.7% mAP@0.5	No
Wang et al. (2025)	ELM-YOLOv8n	96.7% mAP@0.5	No
Gong and Zhang (2023)	YOLOv8 Variant	86.9% mAP@0.5	No
Lv and Su (2024)	YOLOv5-CBAM-C3TR	92.4% Accuracy	No
Scientific (2025)	VGG-DAGSVM	96.5% Accuracy	No
Luo (2025)	AppleLite-YOLOv8	97.56% Precision, 94.38% Recall	No
Rajput et al. (2024)	EfficientNetB0 + Neutrosophic Logic	99.51% Accuracy	No
Parashar and Johri (2024)	CNN + Canny Edge	94.76% Accuracy	No
Rohith et al. (2025)	ResNet + VGG19	98.9% Accuracy (Validation)	No
Liu et al. (2024)	MCDCNet	66.8% Accuracy	No
Zhang et al. (2023)	BCTNet	85.23% Accuracy	No
Khan et al. (2022)	Two-Stage System	88% Classification Accuracy	No
Zhang et al. (2024)	Inc-RPN	49.0% Recall, 34.0% mAP	No
This Study	**LeafSightX (DenseNet201 + InceptionV3 fusion)**	**99.64%**	**Yes**

## 5 Conclusion

This study has led to the development of LeafSightX. This diagnostic framework leverages deep learning to address key challenges in the automated diagnosis of apple leaf diseases, including limited generalizability, interpretability issues, and sensitivity to domain shifts. LeafSightX achieves this by combining features from the DenseNet201 and InceptionV3 backbones using Multi-Head Self-Attention, effectively capturing both detailed and high-level spatial information. A robust preprocessing and augmentation pipeline, along with Grad-CAM visualizations for explanations, further enhances the model's reliability and transparency. Experimental results indicate that LeafSightX delivers outstanding performance, with 99.64% accuracy, an F1-score above 0.996, and perfect AUC and PR-AUC scores, all while maintaining low inference latency, making it suitable for real-time applications in the field. These results surpass multiple baselines and demonstrate the model's consistency across cross-validation splits, as evidenced by a mean Cohen's Kappa of 0.9917 and a standard deviation of 0.0020. Also, the proposed LeafSightX framework was trained and evaluated on an additional independent apple leaf disease dataset, achieving a test accuracy of 99.69%, demonstrating its robustness and generalizability. The broader significance of this research lies in its dual focus on predictive accuracy and model interpretability, meeting the increasing demand for trustworthy AI in agricultural diagnostics. LeafSightX stands as a feasible solution, particularly effective for on-device deployment in underprivileged rural areas. In terms of limitations, LeafSightX was primarily trained on region-specific datasets, which may limit its generalizability to apple leaf diseases in other geographic regions.

Future research will involve further development of LeafSightX as an apple leaf disease detector using large datasets, as a component of edge computing systems, and adaptation to the temporal dynamics of leaf infections through sequential imaging. Finally, LeafSightX will be a more intelligent, explainable instrument to improve control of apple leaf disease in precision agriculture.

## Data Availability

The original contributions presented in the study are included in the article/supplementary material, further inquiries can be directed to the corresponding authors.
